# The N-Terminal Domain of the Repressor of *Staphylococcus aureus* Phage Φ11 Possesses an Unusual Dimerization Ability and DNA Binding Affinity

**DOI:** 10.1371/journal.pone.0095012

**Published:** 2014-04-18

**Authors:** Anindya Biswas, Sukhendu Mandal, Subrata Sau

**Affiliations:** Department of Biochemistry, Bose Institute, Kolkata, West Bengal, India; Florida International University, United States of America

## Abstract

Bacteriophage Φ11 uses *Staphylococcus aureus* as its host and, like lambdoid phages, harbors three homologous operators in between its two divergently oriented repressor genes. None of the repressors of Φ11, however, showed binding to all three operators, even at high concentrations. To understand why the DNA binding mechanism of Φ11 repressors does not match that of lambdoid phage repressors, we studied the N-terminal domain of the Φ11 lysogenic repressor, as it harbors a putative helix-turn-helix motif. Our data revealed that the secondary and tertiary structures of the N-terminal domain were different from those of the full-length repressor. Nonetheless, the N-terminal domain was able to dimerize and bind to the operators similar to the intact repressor. In addition, the operator base specificity, binding stoichiometry, and binding mechanism of this domain were nearly identical to those of the whole repressor. The binding affinities of the repressor and its N-terminal domain were reduced to a similar extent when the temperature was increased to 42°C. Both proteins also adequately dislodged a RNA polymerase from a Φ11 DNA fragment carrying two operators and a promoter. Unlike the intact repressor, the binding of the N-terminal domain to two adjacent operator sites was not cooperative in nature. Taken together, we suggest that the dimerization and DNA binding abilities of the N-terminal domain of the Φ11 repressor are distinct from those of the DNA binding domains of other phage repressors.

## Introduction

The two alternate life cycles (lytic and lysogenic) of temperate bacteriophages are typically regulated by phage-encoded repressor proteins and cognate operator DNAs [1]–[5]. These regulatory proteins typically block the transcription of phage genes from their early promoters by binding to partially overlapped operator DNA sequences. Repressors contain single or multiple domains, exist as monomers or multimers in solution, and possess various masses and sequences. Different temperate phages harbor operator DNAs that have different sizes, sequences and types. Operator DNAs can be composed of sequences with direct or inverted repeats. Repressors also bind to operator DNAs with no repeats. Studies on phage repressors have greatly enriched the fields of both applied and basic molecular biology. Despite their usefulness, most phage repressors have not yet been investigated in detail.

**Figure 1:**
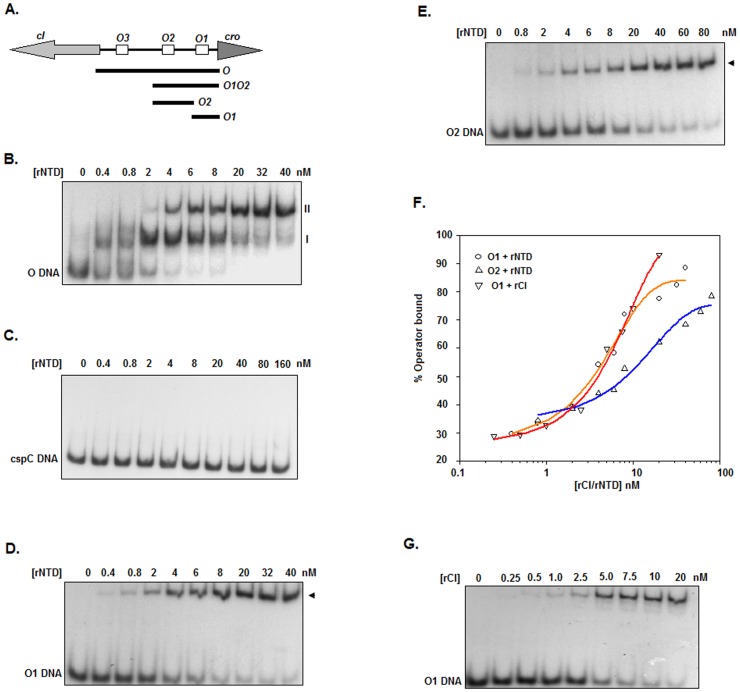
Gel shift assay. (A) Graphic illustration of the Φ11 immunity region (not drawn to scale). The three 15 bp operators (*O1*, *O2*, and *O3*; rectangular boxes) in the *cI-cro* intergenic region, the *cI*- and *cro*-specific open reading frames (divergent arrows), and the DNA fragments (black bars) generated from the immunity region (for gel shift or footprint assays) are shown. (B-E and G) Autoradiograms of gel shift assays with different ^32^P-labeled DNA fragments (shown at the bottom left corner) and the indicated amounts of rNTD or rCI. The shifted complex is represented by either I/II or arrowheads. (F) Plots of the percent of operator bound versus the rNTD or rCI concentration were generated using the data from the scanned autoradiograms shown in panels D, E and G. The curves obtained from the equilibrium binding analysis of rCI to *O1* and rNTD to *O1* or *O2* DNA are presented.

Bacteriophage Φ11, a temperate *Staphylococcus aureus* phage, produces two repressors (CI and Cro) to control its lytic and lysogenic life cycles [1], [6]–[9]. While the lysogenic repressor (CI) seems to be required for the establishment and maintenance of lysogenic development, Cro is suggested to promote the lytic development of Φ11 by inhibiting the expression of CI. The synthesis of CI and Cro occurs from the contiguous *cI* and *cro* genes, respectively, which are situated divergently in the Φ11 genome (Figure 1A). The *cI-cro* intergenic region harbors three 15 bp operator DNA sequences (*O1*, *O2*, and *O3*), which possibly partially overlap with the promoters of the *cI* and *cro* genes [7]–[9]. *O1* and *O3* are positioned upstream of *cro* and *cI*, respectively. Recombinant CI was previously shown to only interact with *O1* and *O2*. In contrast, recombinant Cro exhibits binding only to *O3*. The binding modes of Φ11 repressors do not match with those of lambdoid phages even though the arrangement of the *cI* and *cro* genes in these phages are identical [8], [9]. In addition, the sequences, sizes and spacing of the operator DNAs of Φ11, λ and other related phages are mostly dissimilar. Moreover, *P*_L_-*O*_L_, an additional promoter-operator system that is involved in the lysogenization of phage λ, has yet to be identified in Φ11. Despite the above discrepancy, Φ11 CI is quite similar to λ CI in many ways. The amino acid sequence of Φ11 CI shares 19% identity and 38% positivity to that of λ CI [7]. Φ11 CI is also composed of α-helices and β-sheets, exists as a dimer in solution, cooperatively interacts with *O1* and *O2* and binds to operator DNA as a dimer [8].

Repressor proteins, which are required for the lysogenic development of temperate phages, usually harbor two domains (an N-terminal domain and a C-terminal domain) and a flexible region between the domains [1], [3], [4]. Studies on the domains of several phage repressors revealed many striking similarities and dissimilarities. The C-terminal domains of λ CI, 434 CI, P22 C2, 186 CI, and TP901-1 CI form dimers and, often, other higher order oligomers in solution [10]–[17]. This domain is, therefore, involved in the self-association of these repressors. The C-terminal domain-mediated self-association of λ CI appears to be crucial for the binding of this regulatory protein to adjacent and distant operator sites [1], [18]. The N-terminal domains of λ CI, 434 CI, P22 C2 and TP901-1 CI exist as monomers in solution [10]–[12], [19]. Furthermore, structural studies revealed that the N-terminal domains of both P22 C2 and 434 CI are monomeric in nature [20]–[22]. In contrast, the crystal structure of the N-terminal domain of λ CI showed that it is dimeric in nature [23]. The N-terminal domain of 186 CI also dimerizes weakly in solution but is unable to function *in vivo* [14]. Surprisingly, the N-terminal domain of 186 CI and those of the lambdoid phage repressors possess analogous structures even though their sequences share very little homology [16]. The N-terminal domains of all of the above phage repressors, however, bind operator DNA very weakly [10]–[12], [14], [19]. Structural investigations have revealed that the N-terminal domains of λ CI, 434 CI, and P22 C2 bind their respective operator DNAs as dimers [12], [24]–[27]. Very recently, the N-terminal domain of TP901-1 CI was shown to form a complex with one half of the cognate operator DNA [19]. All structural studies unanimously indicate that the N-terminal domains of phage repressors bind to cognate operator DNA via their helix-turn-helix (HTH) motifs [16], [19], [23]–[25], [28]. The structure-function relationships of the N-terminal domains of most other phage repressors have not yet been demonstrated.

The Φ11 CI monomer carries 239 amino acid residues and is composed of an N-terminal domain (NTD), a C-terminal domain (CTD), and a very small protease-sensitive region between two domains [8]. The CTD shares significant similarity to the C-terminal domains of the *E. coli* LexA and lambdoid phage repressors indicating its participation in the dimerization of Φ11 CI [7], [8]. Conversely, the NTD is likely employed in Φ11 operator DNA binding, as it carries a putative helix-turn-helix (HTH) motif [7]. The tentative HTH motif of the NTD belongs to the HTH-XRE family of transcriptional regulators [7]. Several of these regulators (e.g. XdrA, Stl, PsdR, SinR, etc.) are involved in bacterial pathogenesis [29]–[33]. Currently, little is known about the DNA binding regions of most of these regulators, particularly those belonging to staphylococcal systems. In the present study, we investigated the structure and function of the Φ11 CI NTD using recombinant NTD (rNTD) as a model protein. Our data indicated that the dimerization and operator binding abilities of the N-terminal domain of this Φ11 repressor are significantly different from those of the N-terminal domains of other phage repressors.

## Materials and Methods

### Materials

Acrylamide, bis-acrylamide, DMS (dimethyl sulfate), glutaraldehyde, IPTG (isopropyl β-D-1-thiogalactopyranoside), and PMSF (phenylmethane sulfonylfluoride) were purchased from Sigma (St. Louis, USA), SRL (Mumbai, India) and Merck (Mumbai, India). The alkaline phosphatase-tagged goat anti-mouse antibody (IgG1-AP), anti-His antibody, and Ni-NTA resin were purchased from Santa Cruz Biotechnology Inc. (Dallas, USA) and Qiagen (Hilden, Germany), respectively. The PCR kit, plasmid isolation kit, restriction and modifying enzymes, QIAquick gel extraction kit, oligonucleotides, protein and DNA markers were procured from Qiagen, Hysel India Pvt. Ltd. (New Delhi, India), and Genetix Biotech Asia Pvt. Ltd. (New Delhi, India). [γ-^32^P]ATP was purchased from BARC (Mumbai, India). Growth medium was obtained from Difco Laboratories (Detroit, USA). *E. coli* core RNA polymerase was acquired from Epicenter (Hyderabad, India).

### Bacterial Strains, Plasmid and Oligonucleotides

All of the bacterial strains and plasmids used in this study are listed in Table S1. *Escherichia coli* cells were routinely grown in Luria-Bertani broth supplemented with and without the appropriate antibiotic [34]. *E. coli* strain that harbors a derivative of plasmid pET28 (Novagen, USA) was induced with IPTG [34]. *E. coli* strain BL21(DE3) and plasmid pET28a were obtained as gifts from the late Dr. P. Roy (Bose Institute). All of the oligonucleotides used in this study are listed in Table S2.

### Basic DNA and Protein Methods

Plasmid DNA isolation, polymerase chain reaction (PCR), DNA estimation, DNA treatment with restriction and modifying enzymes, DNA transformation, agarose gel electrophoresis, DNA fragment purification, and DNA fragment labeling with [γ-^32^P]ATP were performed using the standard methods [34], [35] or according to the protocols provided by the respective manufacturers (Qiagen, Fermentas GmbH, and Bangalore Genei Pvt. Ltd.). The sequences of all DNA fragments constructed by PCR were confirmed by DNA sequencing. The total protein content was determined by Bradford assay using bovine serum albumin as the standard [36]. Native, SDS, and urea PAGE, polyacrylamide gel staining and Western blot experiments were carried out as described previously [35].

### Construction of Plasmids and Strains

To express Φ11 CI with a C-terminal histidine-tag (designated rCI), plasmid p1304 (Table S1) was constructed by cloning a DNA fragment (amplified with *Taq* DNA polymerase using pSAU1220 DNA [7] as the template and the primer pairs AN-C1 and AN-C2) into pET28a. To express rNTD, p1315 (Table S1) was generated by cloning a DNA fragment (amplified with *Taq* DNA polymerase using pSAU1220 DNA [7] as the template and the primer pairs NTD1 and NTD2) into pET28a. Strains SAU1304 and SAU1315 (Table S1) were created by transforming *E. coli* BL21 (DE3) with the plasmids p1304 and p1315, respectively.

### Purification of Recombinant Proteins

rCI, rNTD and His-σ^A^ (primary sigma factor of *S. aureus* with a histidine tag) were purified from *E. coli* strains SAU1304, SAU1315, and SAU1283, respectively, using standard methods [7], [37]. Briefly, IPTG-induced cells were ruptured in buffer B [20 mM Tris-HCl (pH 8.0), 300 mM NaCl, 10 mM imidazole, 5% glycerol and 10 µg/ml PMSF], followed by purification of the polyhistidine-tagged recombinant protein (rCI or rNTD or His-σ^A^) from the crude extract by Ni-NTA column chromatography (Qiagen). The eluted proteins were dialyzed against buffer C [10 mM Tris-HCl (pH 8.0), 200 mM NaCl, 1 mM EDTA, and 5% glycerol] for 12–16 h at 4°C. All proteins in buffer C were stored on ice until use. The molar concentrations of the recombinant proteins were determined using the molecular masses of their respective monomeric forms.

### Structural Investigation of Recombinant Proteins

To obtain information about the structures of rCI and rNTD, their far-UV circular dichroism (CD) spectra (200–260 nm) and near-UV CD spectra (250–310 nm) were collected as previously described [38]. The protein concentration in the near-UV CD experiment was 30 µM, and that in the far-UV CD experiment was 10 µM. To estimate the extent of secondary structure in the proteins, all far-UV CD spectra were analyzed with CDNN software [39].

To learn about the oligomeric status of rCI and rNTD in solution, these recombinant molecules (1–5 µM) were respectively crosslinked using glutaraldehyde (0.1%) as previously described [7]. To verify the chemical crosslinking data, analytical gel filtration chromatography of rCI and rNTD (each 10–20 µM) in buffer C (supplemented with 10 mM β-mercaptoethanol) was carried out as reported earlier [7].

### Synthesis and Labeling of DNA Fragments

For gel shift assays, various DNA fragments (*O*, *O1O2*, *O1*, *O2*, and *cspC*) were prepared using a similar procedure to that reported previously [8]. Briefly, the *O* and *O1O2* DNA fragments were synthesized by PCR using pSAU1201 (Table S1) and the primer pairs IIa/pHC1 and pHC1/pHC2, respectively (Table S2). The *O*1 and *O*2 DNA fragments were made by combining and annealing the oligonucleotides PCR11/PCR21 and IIa/IIb, respectively (Table S2). The *cspC* DNA fragment was generated by PCR using *S. aureus* Newman DNA [7] as a template and the primers CSP4 and CSP6. All of the DNA fragments were separated by agarose gel, purified and labeled with [γ-^32^P]ATP as described [8]. With the exception of the *cspC* DNA fragment, all DNA fragments carry one or more Φ11 operator sequence (Figure 1A).

In the DMS footprinting experiment, the labeling of *O* DNA was carried out by two ways as follows. To label the top strand of *O* DNA with ^32^P, *O* DNA was generated by PCR using pSAU1201 DNA, pHC1 and the ^32^P-labeled pHC2. To label the bottom strand of *O* DNA with ^32^P, *O* DNA was PCR amplified using pSAU1201 DNA, ^32^P-labeled pHC1 and pHC2. All of the labeled DNA fragments were separated by agarose gel and purified.

### Gel Shift Assay

To determine the operator DNA binding affinities of rCI and rNTD, the equilibrium binding of these recombinant proteins to different ^32^P-labeled DNAs (*O*, *O1O2*, *O1*, *O2*, *cspC*) was studied by gel shift assay essentially as stated previously [7], [8]. Briefly, different amounts of rCI or rNTD were incubated with ∼0.1 nM ^32^P-labeled DNA on ice for 20 min. After adding loading dye (without SDS), the reaction mixtures were analyzed by native PAGE. The binding of the proteins to *O1* or *O2* were investigated by 10% native PAGE, and the remainder of the DNA-protein interactions were studied by 6% PAGE. The amounts of bound and unbound DNA were determined using the data from the scanned autoradiogram. The *K*_D_ (equilibrium dissociation constant at the half-maximal binding concentration of the protein) values for the interaction between the DNA and protein (rCI or rNTD) were calculated by sigmoidal fitting of the gel shift assay data using Microcal Origin (version 6.0).

To study the cooperative binding activity of rNTD, the binding of varying concentrations of this macromolecule to ^32^P-labeled *O1O2* DNA was investigated by gel shift assay as stated above. The cooperativity parameter was determined from the data from the scanned autoradiogram using standard methods [40].

To gain an understanding of the gene regulatory activity of rNTD and rCI, a different gel shift assay was carried out using a published procedure [37] with modifications. Either rNTD or rCI (50–100 nM) was added to 20 µl buffer C containing 0.1 nM ^32^P *O1O2* DNA and/or RNA polymerase (33 nM His-σ^A^ and 15.4 nM *E. coli* core RNA polymerase). After 20 min of incubation on ice, gel loading dye (without SDS) was added to the reaction mixture, and the assay was performed as described above.

To obtain insight on the binding stoichiometry of rNTD, a gel shift assay was performed using higher rNTD concentrations (0.08–1.6 µM), 0.4 µM cold *O1* DNA, and 0.1 nM ^32^P-labeled *O1* DNA. The rNTD used in the binding stoichiometry experiment and gel shift assay was assumed to possess 80% activity at 0°C.

### DMS Protection Assay

The DMS protection assay was performed as previously reported [8]. rNTD (1 µM) was incubated with 50 nM ^32^P-labeled *O* DNA (∼5000 cpm) in buffer C for 20 min at room temperature. The rNTD-*O* DNA complex was treated with 0.2% DMS for 2 min at room temperature, followed by the termination of the reaction with DMS stop solution [1.5 M sodium acetate (pH 7.0), 1 M β-mercaptoethanol]. Next, the DNA (designated as experimental DNA) was purified from the reaction mixture by passing it through a phenol and chloroform (1∶1) extraction step and then an ethanol precipitation step. Similarly, DNA was purified from DMS-treated, labeled *O* DNA without rNTD (designated as control DNA). G-specific DNA fragments were generated from both the experimental and control DNAs using a standard procedure [41]. The DNA fragments were resolved on a 6% polyacrylamide gel containing urea along with adenosine plus guanine sequencing ladders, which were made from the same labeled *O* DNAs by a standard method [7]. The separated DNA fragments in the gel were visualized using a Typhoon Trio^+^ phosphorimager (GE Healthcare).

### Homology Modeling

To identify a template to develop a three-dimensional model of the NTD, the PDB was searched by PSI-BLAST (http://www.ncbi.nlm.nih.gov/BLAST) using amino acid residues 1-118 of this Φ11 CI domain. The best template (*E* value: 3e-06) was the NMR structure of the C-terminal domain of the Mbf1 protein of *Trichoderma reesei* (PDB ID: 2JVL) [42]. The C-terminal domain of Mbf1 (amino acid residues 29–92) showed 33% identity and 57% positivity with amino acid residues 4-65 of Φ11 CI (data not shown). A structural model of the NTD was generated with Swiss-Model (http://ExPasy.org) using 2JVL (chain A) as the template. Molecular visualization of the resulting model was achieved with Swiss-PDB Viewer (http://ExPasy.org) and PyMol (http://PyMol.org).

### Statistical Analysis

All data are presented as the mean and standard deviation of at least three independent experiments. The mean, standard deviation, and *p* values were calculated using the AVERAGE, STDEV, and TTEST functions of the Microsoft Excel, respectively. Two results were considered significantly different if the related *p* value was <0.05.

## Results and Discussion

### Purification, physicochemical properties and structures of rCI and rNTD

**Figure 2:**
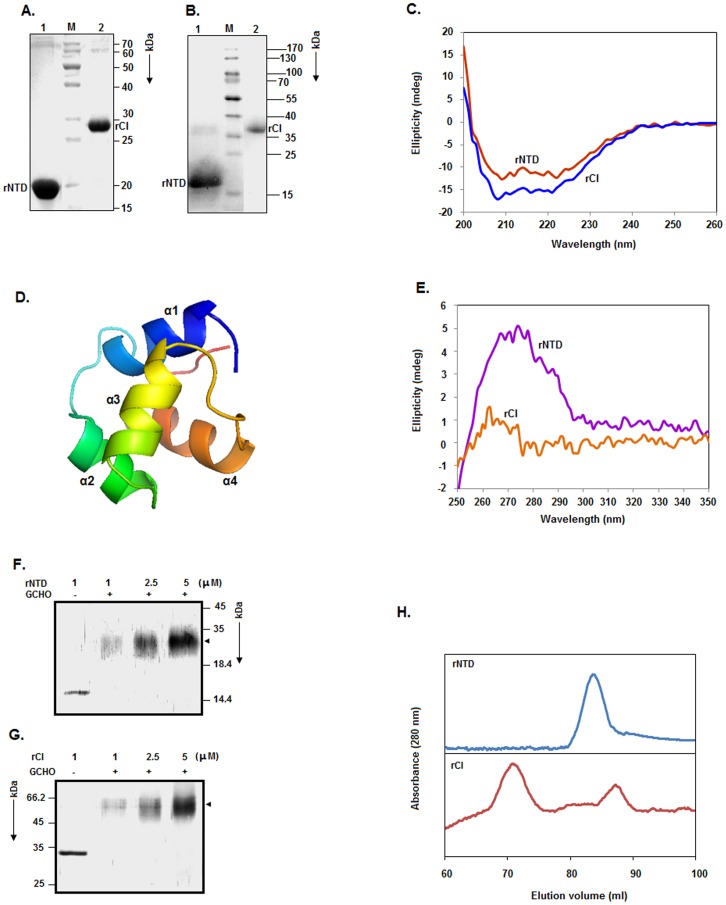
Purification and properties of rNTD and rCI. (A) 13.5% SDS-PAGE analysis of the elution fractions prepared from the SAU1315 (lane 1) and SAU1304 (lane 2) cell extracts. The rNTD- and rCI-specific protein bands are indicated. The molecular masses (in kDa) of the marker protein bands (M) are shown on the right side of the gel. (B) Western blot analysis of the elution fractions prepared from the SAU1315 (lane 1) and SAU1304 (lane 2) cell extracts. The rNTD-, rCI-, and marker protein-specific bands are noted. (C) Far UV CD spectra of rNTD and rCI. (D) Three-dimensional structural model of the N-terminal domain of Φ11 CI. (E) Near UV CD spectra of rNTD and rCI. (F, G) Chemical crosslinking of rNTD and rCI. The proteins were treated separately with glutaraldehyde (GCHO), followed by analysis using 13.5% SDS-PAGE. The arrowheads indicate the dimer-specific protein band. The molecular masses (in kDa) of the marker protein bands are shown on the right or left side of the gels. (H) Gel filtration chromatography of rNTD and rCI by a standard procedure, as described in the Materials and Methods section.

To obtain information on the structure and DNA-binding activity of the NTD of Φ11 CI, we purified recombinant NTD (rNTD) by affinity column chromatography (Figure 2A). rNTD is composed of amino acid residues 1-118 of Φ11 CI and an additional eight amino acid residues (including a stretch of six histidine residues) at its C-terminus. To properly compare the structure and function of rNTD with those of intact Φ11 CI, Φ11 CI with a C-terminal histidine tag (rCI) was designed and purified in similar manner (Figure 2A). Both rNTD and rCI reacted with an anti-his antibody (Figure 2B), and their molecular masses nearly matched with their calculated molecular masses. His-CI (N-terminal histidine-tagged Φ11 CI), which we purified in an earlier work [8], was not used, as it carries 37 additional amino acid residues and its purification protocol is comparatively complex.

To determine the secondary structures of rCI and rNTD, the far-UV CD spectra of these macromolecules were recorded as described in the Materials and Methods section. Figure 2C shows that the spectrum of each protein is composed of two peaks with large negative ellipticity at ∼208 and ∼222 nm, suggesting the presence of an α-helix in each protein. Analysis of the spectra by CDNN [39] suggested that rNTD is composed of ∼33% α-helix, ∼17% β-sheet, and ∼33% coil, whereas rCI carries ∼22% α-helix, ∼23% β-sheet and ∼39% coil. To confirm the above data, we generated a structural model of the Φ11 CI NTD using a template that was different from our earlier template [8]. All of our modeling investigations, however, suggested that a significant proportion of α-helix in CI is contributed by its N-terminal domain, as the NTD is primarily composed of α-helix (Figure 2D).

The near UV CD spectrum (250–350 nm) of a protein usually provides clues about its tertiary structure [43]. This type of spectrum generally originates from the presence of aromatic amino acids, disulfide bonds and cofactors in the protein. Both rNTD and rCI possess all of the aromatic amino acid residues except for Trp. In addition, there are three Cys residues in rCI. To learn about the tertiary structures of rNTD and rCI, we recorded the near UV-CD spectra of these macromolecules as described in the Materials and Methods. Figure 2E shows that the near UV CD spectrum of rNTD drastically differed from that of rCI. While the spectra of rNTD possessed a flattened peak of positive ellipticity at ∼267–280 nm, that of rCI contained a compressed peak of positive ellipticity at ∼260–280 nm. The ellipticity value of the peak in the near UV CD spectrum of rCI was approximately 72% less than that of rNTD. A disulfide bond usually yields a peak at 260 nm, whereas Phe and Tyr result in peaks at ∼255–270 and ∼275–282 nm, respectively [43]. The peak in the spectrum of rNTD, therefore, may have arisen due to the fusion of Phe- and Tyr-specific peaks. In contrast, the origin of the spectrum of rCI seems to be more complicated, as it did not show any clear Tyr-specific peak. The data, however, suggest that rNTD and rCI possess dissimilar tertiary structures. Various factors (such as protein rigidity, hydrogen bonding, polar groups, number of aromatic amino acid residues, interaction between closely situated aromatic amino acid residues, etc.) have been reported to affect the near UV CD spectrum of a protein [43]. The different tertiary structure of rCI may be due to the presence of additional aromatic amino acid residues in this protein (five Phe and five Tyr). In addition, its three Cys residues may also contribute to the near UV CD spectra of rCI.

To determine whether rNTD exists as a monomer or oligomer in aqueous solution, we carried out glutaraldehyde-mediated crosslinking of this protein as described in the Materials and Methods section. Figure 2F shows the formation of a ∼30 kDa protein species in a reaction containing 1–5 µM rNTD in the presence of glutaraldehyde, indicating the dimerization of rNTD in solution. As previously noted for His-CI [8], rCI also formed dimers in solution under similar conditions (Figure 2G).

To verify this chemical crosslinking data, we also analyzed rCI and rNTD by gel filtration chromatography. Figure 2H revealed that rNTD at 10 µM yielded a single peak with an elution volume of 83.5 ml, whereas rCI at the same concentration resulted in two peaks with retention volumes of 70.88 and 87.38 ml. At 20 µM, both rNTD and rCI resulted in peaks with similar elution volumes (data not shown). Using the elution profiles of conalbumin (75 kDa), ovalbumin (43 kDa), carbonic anhydrase (29 kDa), and cytochrome C (12.327 kDa), the apparent molecular mass of rNTD was determined from its elution profile as ∼34.71 kDa. Similar analysis revealed that the two peaks of rCI corresponded to the molecular masses of ∼ 95.11 and ∼25.46 kDa. Sequence analysis showed that the molecular masses of the rNTD and rCI monomers were ∼14.95 and ∼28.22 kDa, respectively. Taken together, we suggest that rNTD solely exists as a dimer in aqueous solution. The relatively early elution of dimeric rNTD may be a result of its extended conformation. In contrast, the two peaks of rCI roughly matched to trimers and monomers in aqueous solution. We ruled out the synthesis of rCI trimers, as our present crosslinking study and the previous studies with His-CI [7] detected dimeric rCI in solution. It is likely that dimeric rCI possesses an enlarged conformation, which led to its early elution at 10–20 µM. Similarly, the later elution of monomeric rCI may be due to its compact conformation.

### Binding of rNTD to the Φ11 *cI*-*cro* Intergenic Region

To determine whether rNTD is capable of binding to the primary immunity region of Φ11 (Figure 1A), we performed a gel shift assay using 0–40 nM rNTD and ^32^P-labeled *O* DNA as depicted in the Materials and Methods. Figure 1B reveals the gradual formation of two shifted complexes (I and II) with increasing concentrations of rNTD. Complexes I and II appeared at ∼0.4 nM and ∼2 nM rNTD, respectively. The intensity of complex I increased steadily up to ∼4 nM rNTD and then dropped dramatically starting at 20 nM rNTD. Conversely, the intensity of complex II was progressively augmented when the concentration of rNTD was increased from 2 to 40 nM. Together, these data indicate the presence of two binding sites for rNTD in the *cI*-*cro* intergenic region.

To understand whether the binding of rNTD to the *cI-cro* intergenic region is specific, a gel shift assay was carried out using 0–160 nM rNTD and ^32^P-labeled *S. aureus cspC* DNA (carrying no Φ11 CI binding site). Figure 1C shows no binding of rNTD *to* the *cspC* DNA, indicating that interaction between this protein and *O* DNA is specific under the study conditions.

To uncover whether rNTD binds to the same operator DNA sites as reported for His-CI [8], additional gel shift assays were carried out using varying concentrations of rNTD and ^32^P-labeled *O1* or *O2* DNA. As anticipated, both *O1* (Figure 1D) and *O2* (Figure 1E) resulted one shifted complex at the rNTD concentrations used in this investigation. The plot of the percentage of operator DNA bound versus the rNTD concentration, which was generated using the data from the scanned autoradiograms (Figures 2D and 2E), revealed that the *K*_D_ values for the interactions between rNTD-*O1* and rNTD-*O2* were 3.13±0.51 and 7.83±0.44 nM, respectively (Figure 1F). These data suggest that the operator DNA binding affinity of rNTD to *O1* is nearly 150% higher than that to *O2* (*p* = 0.003).

To examine whether the operator DNA binding affinity of rNTD differs from that of rCI, we also performed a gel shift assay using 0.25–20 nM rCI and ^32^P-labeled *O1* DNA. The plot of the scanned data from Figure 1G shows that the *K*_D_ value for the interaction between rCI-*O1* was 3.17±0.75 nM (Figure 1F), indicating that both rNTD and rCI possess comparable operator DNA binding affinities (*p* = 0.46).

### Interaction between rNTD and the 15 bp Operator DNAs

**Figure 3:**
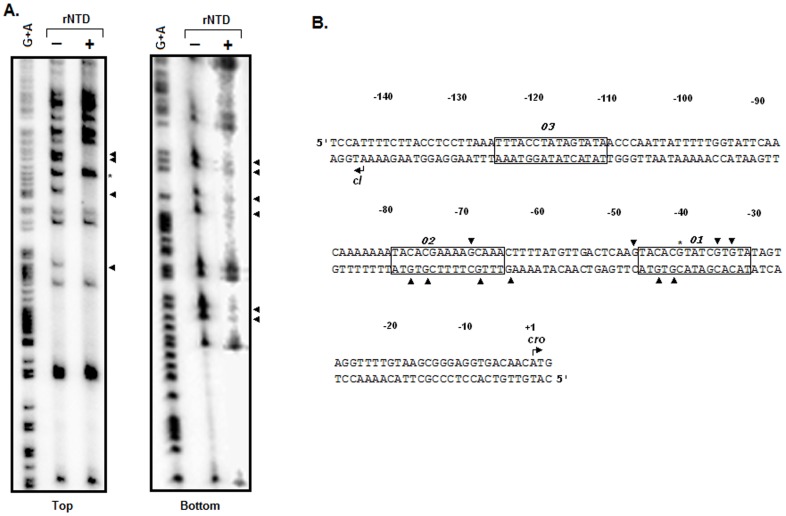
DMS protection assay. (A) Autoradiograms of DMS protection footprints were generated by standard procedures (see Materials and Methods for details) using rNTD (+) or no rNTD (−) and *O* DNA, which was labeled either on the top (Top) or bottom (Bottom) strand. The arrowhead and star indicate the protected guanine (G) base and hypermethylation site, respectively. (B) Summary of the DMS protection footprints. The sequence of the **Φ**11 *cI- cro* intergenic region shows the locations of the three 15 bp *O1*, *O2*, and *O3* sites (solid boxes). The hypermethylated base and protected G bases, identified in panel A, are indicated by arrowheads and stars, respectively. The start codons of Cro and CI are represented by angled arrows. The whole sequence was numbered considering the first base of the Cro start codon as +1.

His-CI was previously reported to interact with a total of eleven G (guanine) bases within the *O* DNA sequence [8]. More precisely, His-CI interacts with four G bases in each of the 15 bp *O1* and *O2* operator DNAs. The remainder of the interacting G bases are located in the spacer region between the 15 bp *O1* and *O2* operator DNAs. To determine whether rNTD interacts with the same G bases in the operator DNA, we performed a DMS protection assay with and without this recombinant domain and ^32^P-labeled *O* DNA. The autoradiograms showed that the intensities of six bottom-strand G bases (-41G, -43G, -63G, -67G, -74G, -76G) and four top-strand G bases (-33G, -35G, -46G, and -68G) in the *O* DNA were remarkably decreased in the presence of a saturating amount of rNTD (Figure 3A). All of the rNTD-protected G bases were situated in and around the 15 bp *O1* and *O2* operators (Figure 3B). These rNTD-interacting G bases have also been shown to exhibit protection with His-CI [8]. The only base that previously showed an interaction with His-CI but was not protected by rNTD was the top-strand base -56G. In addition, the intensity of the top-strand base -40G was notably increased, indicating the increased exposure of this base, which was due to the conformational alteration of the *O* DNA in the presence of rNTD. Interestingly, the intensities of different G bases (-53G on the top strand and -36G and -49G on the bottom strand) are increased when His-CI binds this operator DNA [8]. Despite the minor discrepancy in binding, these results confirm the gel shift assay data (see above) and clearly show that there is an interaction between rNTD and the 15 bp *O1* and *O2* DNAs. Furthermore, like the interaction between His-CI and the operator DNA, the interaction between rNTD and the operator DNA occurs through the major groove of the operator DNA helix, as the N7 of each G base is methylated by DMS and remains exposed in the major groove of the B-DNA helix [1].

### Binding Cooperativity

**Figure 4:**
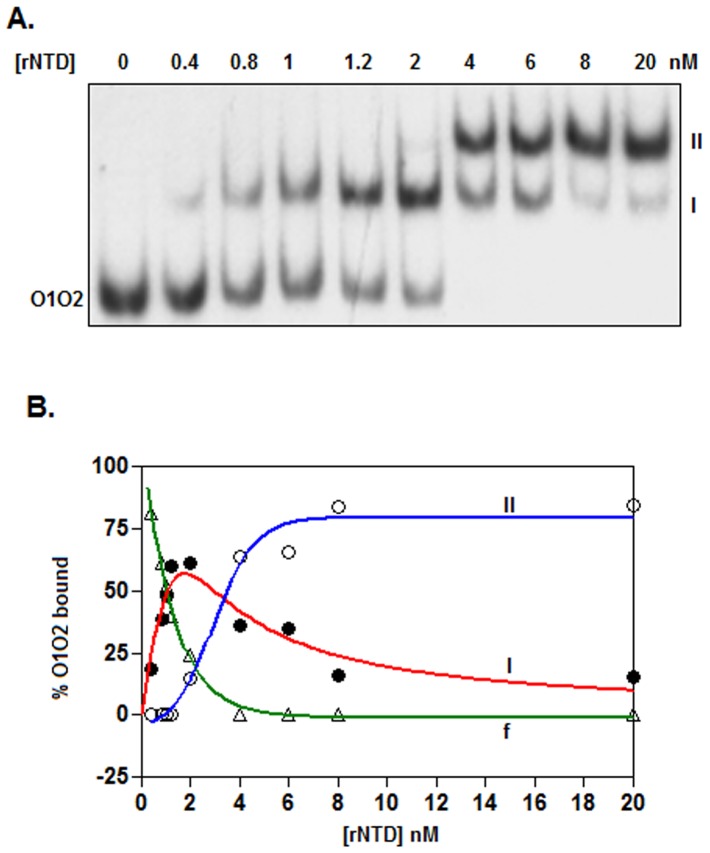
Cooperative binding activity of rNTD. (A) The autoradiogram of the gel shift assay shows the equilibrium binding of rNTD to ^32^P-labeled *O1O2* DNA. Using the data from the scanned autoradiogram, the amounts of unbound *O1O2* DNA and that bound in the shifted complexes (I and II) were determined and plotted against the respective rNTD concentrations. Traces I, II, and f indicate the status of the *O1O2* DNA in complexes I and II and in its unbound state. The data obtained from the traces were analyzed by a standard method as described in the Materials and Methods section. All traces are best-fit traces.

We previously reported that the binding of His-CI to *O1* and *O2* is cooperative in nature [8]. To determine whether dimeric rNTD retains the cooperative binding activity of Φ11 CI, we performed a gel shift assay using varying concentrations of rNTD and ^32^P-labeled *O1O2* DNA as described in the Materials and Methods. The autoradiogram showed two shifted complexes (I and II) in the presence of increasing concentrations of rNTD (Figure 4A). Complex I emerged at 0.4 nM, reached a maximum level at 2 nM and began to decrease at rNTD concentrations of >6 nM. Conversely, complex II was barely detectable at 2 nM, but at 4–20 nM rNTD, it was observed as the major form. The plot of the percentage of *O1O2* DNA bound versus the rNTD concentration (constructed using the data presented in Figure 4A) revealed that complex I contained 61.94±1.33% of the *O1O2* DNA at 2 nM rNTD (Figure 4B). At this rNTD concentration, the amount of *O1O2* DNA in complex II and the amount of the unbound *O1O2* DNA were 14.61±0.13 and 23.45±1.20%, respectively. As demonstrated previously [40], for cooperative binding, there should be less than 50% DNA in complex I and greater than 25% DNA in complex II at 2 nM rNTD. Thus, our results do not suggest the cooperative binding of rNTD to the two 15 bp operators in the *O1O2* DNA fragment.

### Binding Stoichiometry

**Figure 5:**
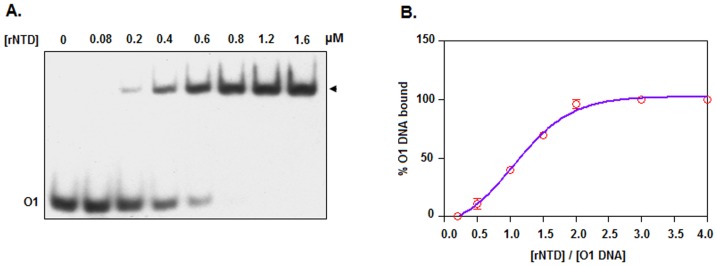
Binding stoichiometry. (A) The autoradiogram of the gel shift assay shows the interaction between the indicated concentrations of rNTD and an excess amount of ^32^P-labeled *O1* DNA (plus excess cold *O1* DNA). Using the data from the scanned autoradiogram, a plot of the percent of bound *O1* DNA versus the rNTD concentration was produced (B).

To determine whether rNTD, like His-CI [8], binds to the 15 bp operator DNA as a dimer, we performed a gel shift assay using very high concentrations of both rNTD and *O1* DNA. To strongly activate the formation of the rNTD-*O1* complex, the concentration of *O1* DNA in this experiment was kept nearly 125-fold above the *K*_D_. Under the conditions stated above, nearly all of the *O1* DNA was shifted at rNTD concentrations of 0.8 µM and higher (Figure 5A). A plot, which was generated using the scanned data from several such gel shift assays, revealed that the rNTD binding was saturated when the ratio of the rNTD concentration to the *O1* DNA concentration reached ∼2.0 (Figure 5B). Together, these data suggest that rNTD, like His-CI [7], binds to the 15 bp operator DNA as a homodimer.

### Effects of rNTD and rCI on the RNA polymerase-*O1O2* DNA complex

**Figure 6:**
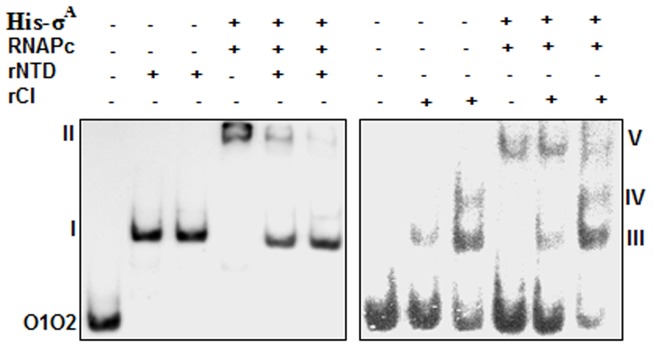
Effects of rNTD/rCI on the DNA binding activity of RNA polymerase. The autoradiograms of the gel shift assay show the interaction between ^32^P-labeled *O1O2* DNA and RNA polymerase [prepared with His-σ^A^ and *E. coli* core RNA polymerase (RNAPc)] in the presence and absence of rNTD or rCI. The shifted bands indicated by II and V were formed by *O1O2* DNA and RNA polymerase. In contrast, the shifted bands indicated by I and III/IV were produced by the binding of rNTD or rCI to *O1O2* DNA.

Our preliminary investigations revealed that there are two divergently oriented promoters in the Φ11 *cI*-*cro* intergenic region. These promoters must be responsible for transcribing two divergently situated Φ11 repressor genes [6]. In λ and other related phages, the binding of repressors to cognate operators prevents the binding of RNA polymerase to overlapped early promoters [1]. To determine whether Φ11 CI and its N-terminal derivative also possess this property, we studied the binding of a heterologous RNA polymerase to labeled *O1O2* DNA in the presence and absence of varying concentrations of rNTD or rCI. The *O1O2* DNA fragment was chosen because it carries the Φ11 *cro* promoter (Biswas and Sau, unpublished observation). Figure 6 shows that the binding of RNA polymerase or rNTD to *O1O2* resulted in one shifted band (II/V or I), whereas the binding of rCI to the same DNA formed two shifted bands (III and IV). Band II/V was most likely generated by the binding of the RNA polymerase to the promoter of the *cro* gene. In contrast, the binding of rNTD or rCI to two operators in *O1O2* produced the bands I, III, and IV. Interestingly, the intensity of band II/V was decreased substantially when rNTD or rCI was added to the reaction mixture. These data suggest that rNTD and rCI affect the binding of RNA polymerase to the Φ11 *cro* gene promoter.

### Effects of Temperature on rNTD

**Figure 7:**
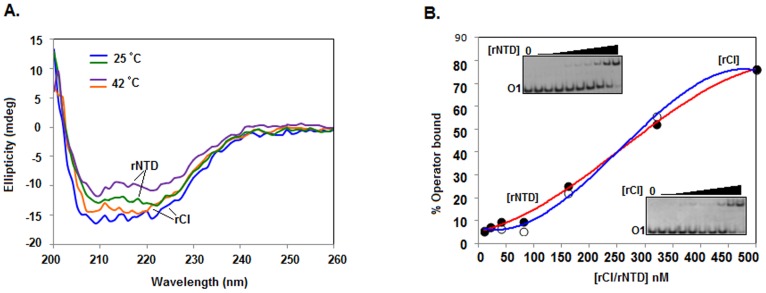
Effect of temperature on rNTD and rCI. (A) Far-UV CD spectra of rNTD and rCI at 25°C and 42°C. (B) Equilibrium binding of rNTD and rCI to *O1* DNA at 42°C. Gel shift assays were performed at 42°C using varying concentrations of rNTD or rCI and 0.1 nM ^32^P-labeled *O1* DNA. Using the data from the scanned autoradiograms (inset), a plot of the percent of bound DNA versus the rNTD or rCI concentration was produced as described in Figure 1.

Increasing the incubation temperature from 25°C to 42°C has been shown to significantly affect both the structure and DNA binding activity of His-CI [7], [8]. As the melting temperature of DNA is higher than 42°C, the structural alteration of His-CI was suggested to be responsible for its reduced DNA-binding affinity at this temperature. To analyze the effect of temperature on the structure and function of the NTD, we first recorded the far-UV CD spectra of rNTD at both 25°C and 42°C. Spectra of rCI were also recorded at 25°C and 42°C for comparison. Figure 7A shows that the peaks in the spectrum of rNTD at ∼208 and ∼222 nm were considerably decreased with the increase in the incubation temperature of this protein from 25°C to 42°C. As shown previously for His-CI [7], the peaks in the spectrum of rCI were also reduced at 42°C. The alteration in the α-helical structure of rNTD at 42°C (Figures 1C and 7A) has also been observed for the DNA-binding domains of other phage-specific repressors [44], [45].

To determine whether the loss of secondary structure in rNTD at 42°C also affects its operator DNA binding activity, the equilibrium binding of this protein was studied with ^32^P-labeled *O1* DNA at 42°C by gel shift assay as described above. The autoradiogram showed that binding of rNTD to *O1* at 42°C was significantly less (Figure 7B, inset) than that observed at 0°C (Figure 1D). The plot of the percentage *O1* DNA bound versus the rNTD concentration (generated using the data from the corresponding autoradiogram) revealed that the *K*_D_ value for the rNTD-*O1* DNA interaction was approximately 300 nM at 42°C. Additional gel shift assays and analyses showed that the *K*_D_ value for the rCI-*O1* DNA interaction was very similar at 42°C (Figure 7B and inset). The DNA binding affinities of rNTD and rCI at 42°C appeared to be nearly 99% less than those at 0°C.

**Figure 8:**
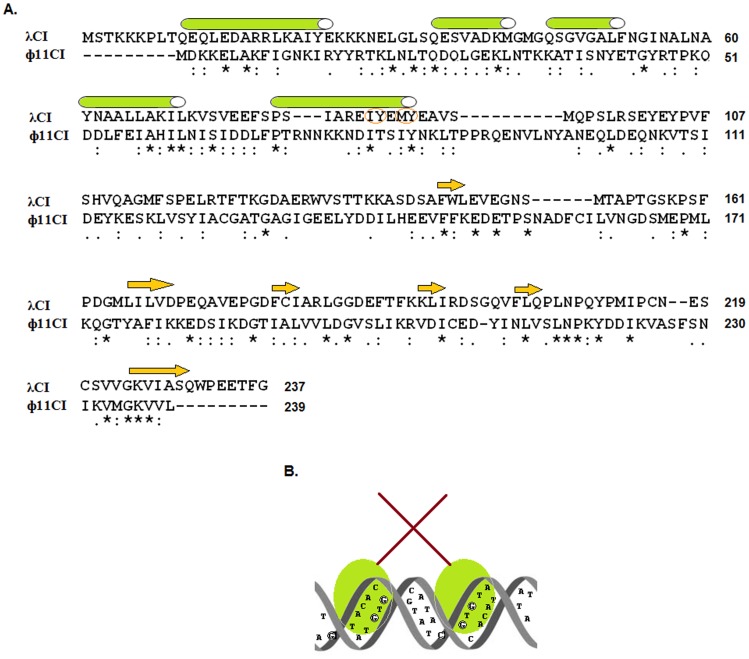
Structure of the *O1* DNA-rNTD complex. (A) The amino acid sequences of a number of λ and Φ11 repressors were aligned using ClustalW. Identical, highly conserved, and weakly conserved residues are indicated by a star, colon and period, respectively. The α-helices and β-sheets, denoted by tubes and arrows, respectively, were adopted from the crystal structure of the N-terminal domain of the λ immunity repressor [23]. The residues involved in the dimerization of the N-terminal domain of the λ immunity repressor [23] are circled. (B) The structural model of the rNTD-*O1* DNA complex, which was generated from our experimental data and analysis, indicates that two domains (formed by amino acid residues 1–61, light green balls) of dimeric rNTD are positioned towards two adjacent major grooves on the *O1* DNA helix. The monomers of the dimeric rNTD contact each other through amino acid residues 62–118 (denoted by a brown rod). The G bases that interact with the dimeric NTDs are circled.

We have demonstrated that rNTD forms homodimers in aqueous solution (Figures 1F and 1G). The N-terminal domains of various phage repressors (e.g., P22 C2, 434 CI, and λ CI) do not form dimers in solution, even at 40–70 µM [10]–[12]. Conversely, the N-terminal domain of the 186 repressor forms dimers in solution, but its dimerization efficiency is approximately 10000-fold less than that of the intact repressor [14]. On the other hand, rNTD formed only dimeric molecules in solution, even at 10 µM (Figure 2G). At equimolar concentrations, rCI existed as both monomers and dimers in solution, indicating that the dimerization ability of rNTD is not only higher than that of rCI but is also unusual in nature. The crystal structure of λ CI NTD was reported to harbor two NTD monomers, each carrying five helices (α1–α5) [23]. While α2 and α3 form the operator-binding HTH motif, α5 participates in the dimerization of λ CI NTD monomers. Four amino acid residues (I85, Y86, M88, and Y89) in the α5 helix of the λ CI NTD monomer are involved in the dimerization of this DNA-binding domain [23]. To identify the residues involved in the dimerization of rNTD and rCI, the amino acid sequence of Φ11 CI was aligned with that of λ CI (Figure 8A). Of the four residues, I85 and Y89 of λ CI aligned with I79 and Y83 of Φ11 CI, whereas M88 of λ CI aligned with I82 of Φ11 CI. These data suggest that I79, Y83, and M88 participate in the dimerization of rNTD. Additional analysis revealed that the region of Φ11 CI that encompasses amino acid residues 62 to 118 carries nearly 72% polar residues, whereas the equivalent region in λ CI harbors only ∼55% polar residues. The excess polar residues in the C-terminal region of rNTD may contribute to the enhanced stability of the rNTD dimer by forming additional ionic/hydrogen bonds. Apart from the hydrophobic and polar residues, multiple H residues at the C-terminal end of rNTD may also have some role in its dimerization. The effects of H residues in this dimerization, however, may be very little, as a histidine tag at the C-terminal end of the N-terminal domain of the phage 186 repressor does not considerably stimulate its dimerization [14].

Our study has demonstrated for the first time that the operator DNA binding affinities of a full-length phage repressor and its N-terminal domain are similar. The degree of the DNA-binding affinity of rNTD has not been observed for the NTDs of the other phage repressors studied to date [10]–[12], [14], [19]. The retention of nearly full DNA binding activity in rNTD may arise from its ability to dimerize with high efficiency. A dimeric repressor usually employs its two DNA binding domains to interact with two adjacent major grooves of an operator DNA helix [1], [4], [23]. Such a complex is stabilized when the non-DNA binding domains of the dimeric repressor also interact with each other. The complex formed between dimeric rNTD and the operator DNA is possibly stabilized via a different mechanism. The region containing amino acid residues 62 –118 in the rNTD monomer likely possesses a self-association property that is similar to that of the C-terminal domain of the Φ11 repressor. As reported for the N-terminal domain of the λ repressor [23], the region encompassing amino acid residues 1–61 in the rNTD monomer may form a globular domain that can interact with a major groove produced by one half of the operator DNA helix. Similarly, a second globular domain from the other monomer of dimeric rNTD may interact with the adjacent major groove generated by the other half of the operator DNA helix. The putative structure of the dimeric rNTD-operator DNA complex (Figure 8B) is supported by two lines of evidence. First, rNTD bound *O1* operator DNA as a dimer (Figure 5). Second, the average size of each DMS-protected region of operator DNA in the presence of rNTD was larger than 10.5 bp (Figure 1B). These results indicate the participation of more than one full turn of B-DNA helix in the interaction with rNTD. Two globular domains of dimeric rNTD, therefore, might have attained a conformation that facilitated the interaction of its two HTH motifs with two adjacent major grooves of the *O1* DNA helix. This conformation also revealed that four G bases (-33G, -35G, -41G and -43G) in *O1* interact with rNTD from the front side of the helix, whereas -46G may do so from the back side of the helix. A similar scheme has been proposed for the interaction between Φ11 CI and the five G bases of *O1* [8].

## Conclusion

Of the domains of Φ11 CI, the NTD is suggested to possess DNA binding activity, as it carries a putative HTH motif. The present investigation revealed that the Φ11 CI NTD exists as a dimer in solution even though the self-association property of CI was assumed to be in its CTD. Surprisingly, the DNA binding activity of the NTD appeared to be similar to that of full-length CI. To the best of our knowledge, the DNA binding domains of no other phage repressors have shown such dimerization and DNA binding abilities. Although they are structurally different, the NTD and full-length CI were both able to bind to two adjacent major grooves of an operator DNA helix. In addition, both proteins affected the binding of RNA polymerase to the operator sites overlapped with the putative phage promoter.

## Supporting Information

Table S1 **Bacterial strains and plasmids used in the study.**
(DOCX)

Table S2 **Particulars of the oligonucleotides used.**
(DOCX)
