# Supplementary material for: The N-Terminal Domain of the Repressor of Staphylococcus aureus Phage Φ11 Possesses an Unusual Dimerization Ability and DNA Binding Affinity
Source: PLoS One. 2014 Apr 18;9(4):e95012. doi: 10.1371/journal.pone.0095012 (PMC3991615; doi:10.1371/journal.pone.0095012)
Supplement: Table S1 — Bacterial strains and plasmids used in the study. (DOCX) [file pone.0095012.s001.docx]

**Supporting information**

**Table S1. Bacterial strains and plasmids used in the study.**

| **Plasmids and Strains Relevant characteristics or genotypes Reference or source** |
| --- |
| Plasmids  pET28a *E. coli*-specific high expression vector, Kan^r^ Novagen  pSAU1201 Plasmid carrying ɸ11 *cI-cro* intergeinc region Das et al., 2007  pSAU1220 pET28a derivative expressing His-CI Das et al., 2007  p1283 pET28a derivative expressing His-σ^A^ Mondal et al., 2010  p1304 pET28a derivative expressing rCI This study  p1315 pET28a derivative expressing rNTD This study  Strains  BL21(DE3) F^-^ *ompT hsdS* (r_B_^-^m_B_^-^) *gal dcm* (DE3) Novagen  XL1 Blue *recA*1 *endA*1 *gyrA*96 *thi hsd*R17 *supE*44  *relA*1 *lac*^-^ F’[*pro*AB+*lacI^q^* *lacZ*∆M15*Tn*10(Tc^r^)] Stratagene  SAU1283 BL21(DE3) harbouring p1283 Mondal et al., 2010  SAU1304 BL21(DE3) harbouring p1304 This study  SAU1315 BL21(DE3) harbouring p1315 This study |
