# Supplementary material for: The N-Terminal Domain of the Repressor of Staphylococcus aureus Phage Φ11 Possesses an Unusual Dimerization Ability and DNA Binding Affinity
Source: PLoS One. 2014 Apr 18;9(4):e95012. doi: 10.1371/journal.pone.0095012 (PMC3991615; doi:10.1371/journal.pone.0095012)
Supplement: Table S2 — Particulars of the oligonucleotides used. (DOCX) [file pone.0095012.s002.docx]

**Supporting Information**

**Table S2.** **Particulars of the oligonucleotides used**

| **Name Sequence (5’-3’) DNA made Reference** |
| --- |
| pHC1 GGATCCTAAATCTTCTTGAGTAC *O* and *O1O2* Das et al., 2007  pHC2 GAATTCTTGGTTCTATAGTATCTG *O* Das et al., 2007  PCR11 gactcaagtacacgtatcgtgtatagt  aggtttA *O1* Das et al., 2007  PCR21 AAACCTACTATACACGATACGTGTACTT  GAGTCA *O1* Das et al., 2007  IIa ATTCAACAAAAAAATACACGAAAAGCA  AACTTTTATGTTGACTCAAGTA *O2* and *O1O2* Das et al., 2007  IIb TACTTGAGTCAACATAAAAGTTTGCTTT  TCGTGTATTTTTTTGTTGAAT *O2* Das et al., 2007  CSP4 CATGCCATGGATGAATAACGGTACAG *cspC* Das et al., 2007  CSP6 CTCGAGCATTTTAACTACGTTTG  *cspC* Das et al., 2007  AN-C1 CATGCCATGGATAAAAAAGAATTAGCG CI This study  AN-C2 CCGCTCGAGCAATACAACTTTGCCCATTAC CI This study  NTD1 CATGCCATGGATAAAAAAGAATTAGCG NTD This study  NTD2 CCGCTCGAGTTTAGACTCTTTATATTCATC NTD This study |
